# Correction: Addison, C.C., *et al*. Psychometric Evaluation of a Coping Strategies Inventory Short-Form (*CSI-SF*) in the Jackson Heart Study Cohort. *Int. J. Environ. Res. Public Health* 2007, *4*, 243–249.

**DOI:** 10.3390/ijerph6030941

**Published:** 2009-03-02

**Authors:** Clifton C. Addison, Brenda W. Campbell-Jenkins, Daniel F. Sarpong, Jeffery Kibler, Madhu Singh, Patricia Dubbert, Gregory Wilson, Thomas Payne, Herman Taylor

**Affiliations:** 1 Jackson Heart Study Coordinating Center, Jackson, MS, USA; 2 Nova Southeastern University, Pensacola, FL, USA; 3 Tougaloo College, Tougaloo, MS, USA; 4 Veterans Administration Medical Center, Jackson, MS, USA; 5 University of Mississippi Medical Center, Jackson, MS, USA; 6 Jackson Heart Study, Jackson, MS, USA

We found some errors in Table 4 in our paper published in the *International Journal of Environmental Research and Public Health* recently [1]. Table 4 is corrected as follows:

**Table 4.** The Survey Item Factor Loadings for the *CSI-SF.*

CSI-SF Survey Items	Mean	SD	Factor*	Loadings
1. I make a plan of action and follow it	3.65653	0.94486	2	**0.92**
2. I look for the silver lining or try to look on the bright side of things	4.09182	0.87564	2	**1.00**
3. I try to spend time alone	3.33692	0.91270	4	**0.88**
4. I hope the problem will take care of itself	2.55667	1.03334	3	**0.93**
5. I try to let my emotions out	3.24203	0.95468	1	**1.00**
6. I try to talk about it with a friend or family	3.55859	0.99147	1	**0.91**
7. I try to put the problem out of my mind	2.97849	1.04488	3	**1.00**
8. I tackle the problem head on	3.50519	0.96533	2	**0.71**
9. I step back from the situation and try to put things into perspective	3.67576	0.90556	2	**0.92**
10. I tend to blame myself	2.59278	1.00314	4	−**0.99**
11. I let my feelings out to reduce the stress	3.24357	0.97027	1	**0.94**
12. I hope for a miracle	3.38110	1.21038	3	**0.90**
13. I ask a close friend or relative that I respect for help or advice	3.34076	0.98582	1	**0.92**
14. I try not to think about the problem	2.91010	0.96179	3	**0.98**
15. I tend to criticize myself	2.59278	1.05723	4	**1.00**
16. I keep my thoughts and feelings to myself	3.02190	0.99340	4	**0.13**

*Factor 1 = Emotion-Focused Engagement, Factor 2 = Problem-Focused Engagement, Factor 3 = Problem-Focused Disengagement, Factor 4 = Emotion-Focused Disengagement

**Responding Scores (1 = never, 2 = seldom, 3 = sometimes, 4 = often, 5 = almost always)

We apologize for any inconvenience caused to the readers.
